# Adolescent Participation in Research, Policies and Guidelines for Chronic Disease Prevention: A Scoping Review Protocol

**DOI:** 10.3390/ijerph17218257

**Published:** 2020-11-09

**Authors:** Mariam Mandoh, Seema Mihrshahi, Hoi Lun Cheng, Julie Redfern, Stephanie R. Partridge

**Affiliations:** 1 Westmead Applied Research Centre, Faculty of Medicine and Health, The University of Sydney, Westmead, NSW 2145, Australia; julie.redfern@sydney.edu.au (J.R.); stephanie.partridge@sydney.edu.au (S.R.P.); 2 Department of Health Systems and Populations, Macquarie University, Macquarie Park, Sydney, NSW 2109, Australia; seema.mihrshahi@mq.edu.au; 3 Sydney Medical School, Faculty of Medicine and Health, The University of Sydney, Discipline of Child and Adolescent Health, Westmead, NSW 2145, Australia; helen.cheng@health.nsw.gov.au; 4 The Children’s Hospital at Westmead, Academic Department of Adolescent Medicine, Westmead NSW 2145, Australia; 5 The George Institute for Global Health, The University of New South Wales, Camperdown, NSW 2006, Australia; 6 Prevention Research Collaboration, Charles Perkins Centre, Sydney School of Public Health, Faculty of Medicine and Health, The University of Sydney, Camperdown, NSW 2006, Australia

**Keywords:** youth, adolescent, engagement, participatory action, health policy, decision-making, chronic disease, non- communicable diseases (NCD), prevention

## Abstract

Adolescents (10–24 years old) account for 23% of the global population. Physical inactivity, suboptimal dietary intake, overweight, and obesity during adolescence are risk factors associated with chronic disease development into adulthood. Research, policies, and guidelines that seek to prevent chronic disease risk factor development rarely engage adolescents in planning and decision-making processes. The aims of this review are to investigate (i) how adolescents currently participate in research, policy, and guidelines for reduction of chronic disease risk factors, and (ii) provide recommendations to optimize adolescent participation in future research, policy, and guideline decision making for chronic disease prevention. A systematic scoping review of the health peer-review research, policy, and guidelines, using Arksey and O’Malley’s six-stage framework, will be conducted. Participatory outcomes will be assessed based on the Lansdown-UNICEF conceptual framework for measuring adolescent participation. Classified as consultative, collaborative, or adolescent-led according to the degree of influence and power adolescents possess in the decision- making processes. Consultation with adolescents via digital surveys and focus groups will provide further information, perspective, and insight. Qualitative data will be analyzed by descriptive numerical summary and qualitative content analytical techniques. The title of this protocol is registered with Joanna Briggs Institute and Open Science Framework, doi:10.17605/OSF.IO/E3S64.

## 1. Introduction

Adolescence is a unique stage of life connecting childhood to adulthood, characterized by rapid cognitive development and accelerated physical growth [1,2]. In conjunction with physiological changes, adolescents exhibit increased autonomy, identity formation, and the development of critical social skills, knowledge and networks that enable them to engage with broader society [3,4,5]. Adolescence plays a critical role in laying the foundations necessary for the development of a productive future workforce, and the next generation of decision makers and leaders [6,7,8]. Today rapid social and technological transformations are directly impacting the understanding of the duration of adolescence [9]. It is now recognized that adolescence encompasses a longer portion of the life course than in any other time in history (10 to 24 years of age) [9]. It is therefore imperative that adolescents are empowered in the decision-making process to make informed health choices at this life stage in order to improve their health and quality of life trajectory.

Chronic disease risk factors, such as physical inactivity, suboptimal dietary behaviors, and overweight or obesity often cluster in adolescence. In 2018 the World Health Organization (WHO) reported that 81% of adolescents aged 11–17 years are insufficiently physically active [10]. Adolescents display a simultaneous increase in obesogenic dietary behaviors as they progress through adolescence, such as reduced fruit and vegetable intake and increasing intake of sugar sweetened beverages [11,12,13].

The association between physical activity, diet, overweight, and obesity in adolescence and chronic disease outcomes as well as quality of living in later life has been well established [14,15,16,17,18]. Seventy percent of preventable adult deaths from chronic diseases are linked to lifestyle risk factors that start during adolescence [19]. Moreover, an inequitable distribution of chronic disease risk factors exists, with a higher prevalence of overweight and obesity among adolescents of low-socioeconomic status in both developing and developed countries [20,21,22,23]. These phenomena are of paramount concern for not only the adolescents themselves and their families, but also governments who face lost productivity associated with chronic diseases as well as an increased strain on health systems to manage chronic diseases. A shift in paradigm of approaches to prevent chronic disease in adolescents is required [24].

The United Nations Convention on the Rights of the Child (UN CRC), declares that “Governments should ensure that children survive and develop healthily” and “Children have the right to good quality health care, clean water, nutritious food, and a clean environment so that they will stay healthy.” The UN CRC also maintains that “participation” in decision-making that affects the life of an adolescent is their legal right [25,26]. The UN CRC treaty has been ratified by all except one country globally [27]. Most participating countries have not achieved their obligations. For example, adolescents are rarely engaged in the health and medical research cycles that shape the chronic disease prevention interventions, guidelines, and policies that serve them [28,29,30]. A 2020 report by the WHO-UNICEF-Lancet Commission addressed the lack of effective and cohesive measures taken by governments globally to tackle adolescent engagement issue successfully [31].

Ineffectively engaging and empowering adolescents in research about their health and the health of their peers will potentially result in unsustainable and unsuccessful chronic disease prevention interventions, policy, and guideline development. This will subsequently result in adverse long-term health consequences for adolescents and their future offspring [31]. The importance of consumer and community engagement in research is widely acknowledged [26,32]. Adolescents represent a key stakeholder group [33], with untapped potential to benefit societies socially, politically, and economically. When given a voice and opportunity adolescents have demonstrated considerable value for meaningful contributions to issues of importance to them, their community and beyond [29]. Still, current evidence suggests that adolescent participation in research is often ad hoc and tokenistic [34].

Definitions of participation in health research vary considerably. Common terminology includes but are not limited to youth-led participatory action research (Y-PAR), community-based participatory research (CBPR), participatory action research (PAR), e-PAR (technology-based PAR), action research (AR), participatory practices, youth engagement, and youth involvement. Furthermore, systematic reviews on participatory methods in adolescent populations vary largely in their scope, in terms of context, age range, demographics or outcomes measured [35,36,37,38,39,40,41]. For example, some reviews are specific in their inclusion criteria of age such as “0–18 years” [36] while other reviews investigate broad definitions of “youth” or “child” participation [40] with health outcomes infrequently evaluated. Adolescent health is a fundamental element necessary to promoting better quality of life and reduced chronic disease risk across the life course. Yet, despite extensive focus on youth participation methods more broadly [42,43], there remains limited research demonstrating effective strategies for adolescents’ participation in research and interventions for chronic disease prevention.

The current body of chronic disease prevention research is lacking in effective and inclusive research, policies, and guidelines which support all adolescents to improve chronic disease risk factors and lead healthy lives [18]. There is a need to synthesize the peer review and grey literature to evaluate adolescents as a unique demographic and assess their current role in health research, policy, and guideline development for chronic disease prevention. The aims of this scoping review protocol are two-fold. First, to conduct a scoping review to analyze the mode of adolescent participation in health peer-reviewed primary research studies, policies, and guidelines for co-designing or decision making for chronic disease risk factor reduction, specifically, physical activity, diet, overweight, and obesity research. Second, to determine, through adolescent participation, recommendations for the most optimal modes of participation for research, policy, and guideline decision- making for chronic disease prevention.

## 2. Materials and Methods

### 2.1. Protocol Design

The approach to be taken to systematically review the knowledge base on our topic of interest was determined by considering the purpose of this review and nature of the data sought. It was determined that a systematic scoping review would best address the study aims. A scoping review is a valid method to broadly “map” the existing literature on a particular topic. For the purpose of this review, it will permit for a broad and thorough examination of the emerging field of research into adolescent participation in research, policies, and guidelines for chronic disease prevention. This will allow for the identification and analysis of key outcomes and knowledge gaps on this topic, whereas a traditional systematic review is narrower in scope, guided by an explicit research question with the purpose to inform clinical practice or policy [44,45].

**Figure 1:**
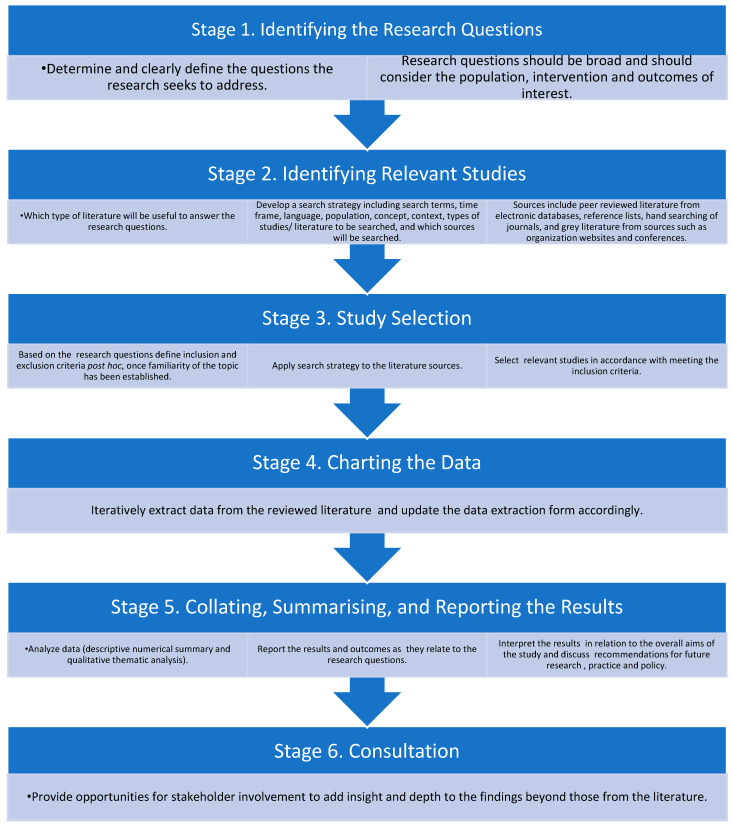
Methodological framework for conducting a scoping review adapted from the Arksey and O’Malley six stage framework.

The scoping review will be informed by Arksey and O’Malley’s six stage framework for scoping reviews [46], with Levac et al.’s recommendations (Figure 1) [45,47]. This framework enables a flexible and iterative approach to systematically scope and interpret a range of peer-reviewed primary research studies and grey literature. The Arksey and O’Malley framework is a published methodology for scoping reviews, which has been further advanced and iterated since inception [47,48]. This framework provides the foundation for the Joanna Briggs Institute (JBI) manual for evidence synthesis scoping review guidelines [45] and provides clear guidance at each stage of the scoping review process to ensure a clear and rigorous review process.

The Preferred Reporting Items for Systematic reviews and Meta-Analyses extension for Scoping Reviews (PRISMA-ScR) checklist [49] will be used to ensure the scoping review meets necessary quality standards of practice and reproducibility.

#### 2.1.1. Identifying the Research Questions

Preliminary search of the peer-reviewed primary research studies and grey literature aided in refining the research questions. The research questions aim to provide evidence and critical insight into how adolescents can effectively participate in co-designing, and in decision making related to research, guidelines, and policies for chronic disease prevention. The main chronic disease risk factors to be included are physical inactivity, suboptimal diet, overweight, and obesity, given their prevalence in adolescence and similarities in primary prevention approaches. The research questions are (i) is there evidence of effectiveness of adolescent participation in reducing chronic disease risk factors in peer-reviewed primary research studies, policies, and guidelines? (ii) what are the components, processes, or conceptual frameworks of effective peer-reviewed primary research studies, policies, and guidelines involving adolescent participation in chronic disease risk factor reduction? and (iii) are there any identifiable barriers or facilitators or evidence gaps for adolescent participation in the chronic disease prevention space?

#### 2.1.2. Identifying Relevant Studies and Study selection

Peer-reviewed primary research studies, policies, and guidelines for inclusion will be identified by systematic and comprehensive searches of electronic health and medical research databases as well as grey literature databases and registers. First, to identify primary research studies, a systematic search of the peer-reviewed literature using medical subject headings (MeSH) terms, keywords, and database categorization will be conducted. This iterative process will involve broad search terms, including combinations, truncations, and synonyms of “nutrition,” “diet,” “physical activity,” “overweight,” “obesity,” “lifestyle risk factors,” “non-communicable diseases,” “adolescents,” “youth,” “teenagers,” “guidelines,” “co-design,” “decision-making,” “youth engagement,” “youth involvement,” “participatory research” (will cover Y-PAR, CBPR, PAR, e-PAR), “action research” and “participatory practices.” Second, to identify relevant policies and guidelines, grey literature sources will be searched using the keywords mentioned above as well as the population (adolescence, youth), concept (adolescent engagement, participation), and context (chronic disease prevention) classification where necessary.

#### 2.1.3. Search Strategy

As recommended by the Joanna Briggs Institute (JBI) for scoping reviews, a three-step search strategy will be utilized. Both peer-reviewed published primary research studies and grey literature will be evaluated. First, a preliminary search of Medline (through PubMed) and Cumulative Index of Nursing and Allied Health Literature (CINAHL) databases will be conducted. Key search terms will be refined by analysis of keywords contained in the title and abstract of the retrieved articles, and of the index terms used to describe the articles.

Second, a systematic and comprehensive search of the peer-reviewed literature will be conducted. All identified keywords and index terms will be searched across all included databases. Six major databases will be examined: Medline (through PubMed), Embase, the Cochrane Central Register for Controlled Trials (CENTRAL), CINAHL, Global health and Scopus. In addition, grey literature will be consulted from the National Health and Medical Research Council (NHMRC) Guideline Index (Australia), the National Guideline Clearinghouse (U.S), the Disease Prevention and Control Guidelines (Canada), the National Institute for Health and Care Excellence-Evidence Search (UK), and the United Nations (UN). A universal internet search engine (Google) advanced search function will be utilized to identify additional polices or guidelines. The grey literature search will be conducted in incognito mode on the search engine to restrict ads and cookies. The search results will be limited to the first five pages of search. The final grey literature source will be Tripdatabase.com which will be examined using the population, concept, context (PCC) search option as described above.

Next, the database results will be pooled, and duplicates removed. A two-part study selection process will be applied to minimize opportunity for errors and reviewer bias. In part one, a title and abstract review will take place by one reviewer (MM), articles that do not meet defined inclusion criteria will be excluded. Part two will involve a full text review of remaining articles by two reviewers (MM and SRP) independently. Articles that do not meet inclusion criteria by both reviewers will be excluded. In cases of disagreement a third reviewer (JR) will adjudicate the final decision.

Finally, reference list examination and citation chaining (both forward and backward where necessary) will be conducted for full text articles that meet all the inclusion criteria as well as those of any identified reviews. Duplicates will be removed and studies that are found via this method will likewise undergo a two-part selection process (as above) to determine eligibility for inclusion in the final scoping review.

### 2.2. Inclusion Criteria

Peer reviewed and grey literature will be subject to inclusion and exclusion criteria defined after preliminary searching, agreed upon by study coordinators. Details of the criteria on which the scoping review will be based are outlined below in accordance with the Joanna Briggs’s Institute (JBI) Manual for evidence synthesis [45]. The population, concept, context, types and sources of literature of interest are defined.

#### 2.2.1. Population

The WHO defines an “adolescent” as a person aged between 10–19 years old, and “youth” as a person aged between 15 and 24 years old [3]. WHO also recognizes that “youth” is a fluid term that varies depending on country and experience [33]. The Lancet Child and Adolescent Health describes an adolescent as one between the age of 10–24 years [9]. Within the published peer-reviewed literature these two terms are used loosely and interchangeably. For a comprehensive analysis of the peer-reviewed literature this scoping review will assess published and grey literature involving “adolescents” or “youth” defined as ages 10–24.

#### 2.2.2. Concept

The fundamental concept to be examined and considered for inclusion is all forms of adolescent participation, engagement, and decision-making in peer-reviewed primary research studies, policy, and guideline development related to physical activity, dietary behaviors, overweight, obesity, and chronic disease prevention. This will include but is not limited to Y-PAR, CBPR, PAR, e-PAR, AR, participatory practices, youth engagement, and youth involvement.

#### 2.2.3. Context

The review will encompass a broad context. All genders and all languages with the exclusion of papers without an abstract published in English will be considered. Published peer-reviewed literature from all countries will be examined, while policies and guidelines will be limited to those from Australia, the United Kingdom (UK), the United States of America (USA), Canada, and the United Nations (UN), reflecting similar demographics and health ethos. Material published prior to 1995 will be excluded to reflect literature, policies, and guidelines likely to impact the current generation of youth, the Generation Y (or “millennials,” born between 1981 and 1996) and Generation Z (born between 1995 and 2015). These generations have in common that they are more technologically savvy and more “connected” than the generations before them [50]. Constant internet access and accessibility of devices have made these generations more conscious of social, environmental, and global political issues than previous generations. This demographic is also unique in that they solve problems in dynamic and innovative ways, they value being involved in decision making and disengage when decisions are imposed upon them, challenging traditional norms [51].

#### 2.2.4. Types and Sources of Literature

This scoping review seeks to capture the current state of adolescent participation in the chronic disease prevention space. Published peer reviewed primary studies, guidelines, and policies are the documents which have the most impact on the chronic prevention processes. These documents are accepted by academic professionals and policy makers and are the basis for driving future program development. Therefore, all published peer-reviewed literature of primary studies as well as published policies and guidelines involving adolescent participation in chronic disease prevention will be assessed for inclusion.

#### 2.2.5. Charting the Data

Interest in measuring and evaluating meaningful youth engagement in projects for research is not a new concept. In 1969 Arnstein developed a “ladder” [52] to metaphorically represent and categorize citizen participation in projects [53]. In 1992 Roger A. Hart, in association with the United Nations Children’s Fund developed the “ladder of participation” (also known as “HART’s ladder”), which involved eight levels or rungs of the ladder, each representing a differing level of young people’s participation [54]. Furthermore, in 2001 Harry Shier proposed an alternative model, “Pathways to participation,” which involved 5 levels of children’s participation and 15 questions spread throughout the pathway. Shier recognized that it is possible to be at more than one stage of the pathway at any one given time. Hence, it does not completely replace Hart’s ladder but acts as an adjunct [53].

More recently, Gerison Lansdown (2018), in association with UNICEF, further built on these models and developed a more comprehensive “Conceptual Framework for Measuring Outcomes of Adolescent Participation,” comprising three modes of meaningful participation: collaborative, consultative, and adolescent-led. This framework, instead of using an approach based on “stages,” emphasizes that true participation which leads to empowerment and influence can be achieved through differing approaches which are valid but suitable for different contexts and subject to evolving capacities [55].

The data will be charted by categorizing the qualitative data according to key concepts using the Lansdown UNICEF (2018) model. Two researchers (MM and SRP) will extract the data to ensure categories are consistent with the research questions. The data extraction form will be iteratively updated and fine-tuned during data collection to ensure all relevant data are obtained.

#### 2.2.6. Collating, Summarizing and Reporting the Results

Data will be analyzed by descriptive summary using a scale to assess research, policies, and guidelines against the key concepts and by qualitative content analytical techniques. The results will be reported considering gaps in the evidence for adolescent engagement in co-design and decision making related to chronic disease prevention and the implications of the findings will be considered within the broader context of adolescent health and non-communicable chronic disease prevention. Since, the results of this scoping review are essentially qualitative, the results will be discussed in narrative form.

#### 2.2.7. Participatory Consultation Exercise

Stage six of the Arksey and O’Malley framework involves a participatory consultation exercise with adolescent stakeholders. This is necessary to provide further depth and context to the results obtained from the literature. This process will be informed by the results of the scoping of published and grey literature.

## 3. Timeline

Our research is in progress. A draft of the scoping review is currently underway and will be submitted before the end of 2020.

## 4. Discussion

Although systematic in nature, this scoping review is not without potential challenges. Despite a comprehensive search, relevant literature could potentially be missed due to terminology differences within this field. Nonetheless, this review will present a summary of adolescent participation in the chronic disease prevention space. It will identify gaps in the literature and specifically analyze the current state of adolescent participation in the health and medical research cycle as well as policy and guideline development processes. This research will provide new insight and solutions to effectively tackle chronic disease risk factor development in communities of low, middle, and high socioeconomic status and varying demographics. Findings will inform advanced resource development, providing a robust foundation for innovative chronic disease prevention research, policy, and guideline development. Electronic resources and tools will be developed and form a tool kit to guide and support researchers, industry, and policy makers on how to effectively engage adolescents at all stages of the research, policy, and guideline development process. This will promote efficient and effective interventions to support the adoption of healthy lifestyle behaviors during adolescence and subsequently reduce the risk of chronic disease onset throughout the life course.

## 5. Conclusions

Youth participation in the research, policy, and guideline development and decision-making processes for chronic disease prevention is a novel approach to the global challenge of chronic disease. By partnering with adolescents to address the root causes of chronic disease risk factor development, this research will aid in finding solutions to overcome facets of chronic disease burden.
